# Degradation of Components of the Lpt Transenvelope Machinery Reveals LPS-Dependent Lpt Complex Stability in *Escherichia coli*

**DOI:** 10.3389/fmolb.2021.758228

**Published:** 2021-12-22

**Authors:** Alessandra M. Martorana, Elisabete C. C. M. Moura, Paola Sperandeo, Flavia Di Vincenzo, Xiaofei Liang, Eric Toone, Pei Zhou, Alessandra Polissi

**Affiliations:** ^1^ Dipartimento di Scienze Farmacologiche e Biomolecolari, Università Degli Studi di Milano, Milan, Italy; ^2^ Department of Chemistry, Duke University, Durham, NC, United States; ^3^ Department of Biochemistry, Duke University School of Medicine, Durham, NC, United States

**Keywords:** bacterial cell envelope, lipopolysaccharide, Lpt system, outer membrane stability, LpxC inhibitor

## Abstract

Lipopolysaccharide (LPS) is a peculiar component of the outer membrane (OM) of many Gram-negative bacteria that renders these bacteria highly impermeable to many toxic molecules, including antibiotics. LPS is assembled at the OM by a dedicated intermembrane transport system, the Lpt (LPS transport) machinery, composed of seven essential proteins located in the inner membrane (IM) (LptB_2_CFG), periplasm (LptA), and OM (LptDE). Defects in LPS transport compromise LPS insertion and assembly at the OM and result in an overall modification of the cell envelope and its permeability barrier properties. LptA is a key component of the Lpt machine. It connects the IM and OM sub-complexes by interacting with the IM protein LptC and the OM protein LptD, thus enabling the LPS transport across the periplasm. Defects in Lpt system assembly result in LptA degradation whose stability can be considered a marker of an improperly assembled Lpt system. Indeed, LptA recruitment by its IM and OM docking sites requires correct maturation of the LptB_2_CFG and LptDE sub-complexes, respectively. These quality control checkpoints are crucial to avoid LPS mistargeting. To further dissect the requirements for the complete Lpt transenvelope bridge assembly, we explored the importance of LPS presence by blocking its synthesis using an inhibitor compound. Here, we found that the interruption of LPS synthesis results in the degradation of both LptA and LptD, suggesting that, in the absence of the LPS substrate, the stability of the Lpt complex is compromised. Under these conditions, DegP, a major chaperone–protease in *Escherichia coli*, is responsible for LptD but not LptA degradation. Importantly, LptD and LptA stability is not affected by stressors disturbing the integrity of LPS or peptidoglycan layers, further supporting the notion that the LPS substrate is fundamental to keeping the Lpt transenvelope complex assembled and that LptA and LptD play a major role in the stability of the Lpt system.

## Introduction

Gram-negative bacteria are surrounded by two distinct lipid bilayers: the inner membrane (IM) and the outer membrane (OM). The IM and OM together delimit an aqueous compartment called periplasm which contains the peptidoglycan (PG), a continuous polymer that protects the cell from osmotic lysis. The outermost layer of the cell envelope, the OM, endows it with highly selective permeability properties (Silhavy et al., 2010). The asymmetric configuration of the OM, with phospholipids in the inner leaflet and lipopolysaccharide (LPS) in the outer leaflet, greatly hampers the permeation of many toxic molecules into the bacterium. The complex architecture of the Gram-negative bacterial cell envelope poses a great challenge to the development of novel antimicrobial molecules (Nikaido, 2003; Tacconelli et al., 2018). Therefore, the disruption of the LPS biosynthetic pathway has long been explored by pharmaceutical companies (Erwin, 2016). Over the last decade, targets in the LPS transport pathway have emerged providing innovative approaches for antibiotic development (Srinivas et al., 2010; Werneburg et al., 2012; Andolina et al., 2018; Vetterli et al., 2018; Moura et al., 2020; Zhang et al., 2019).

**FIGURE 1:**
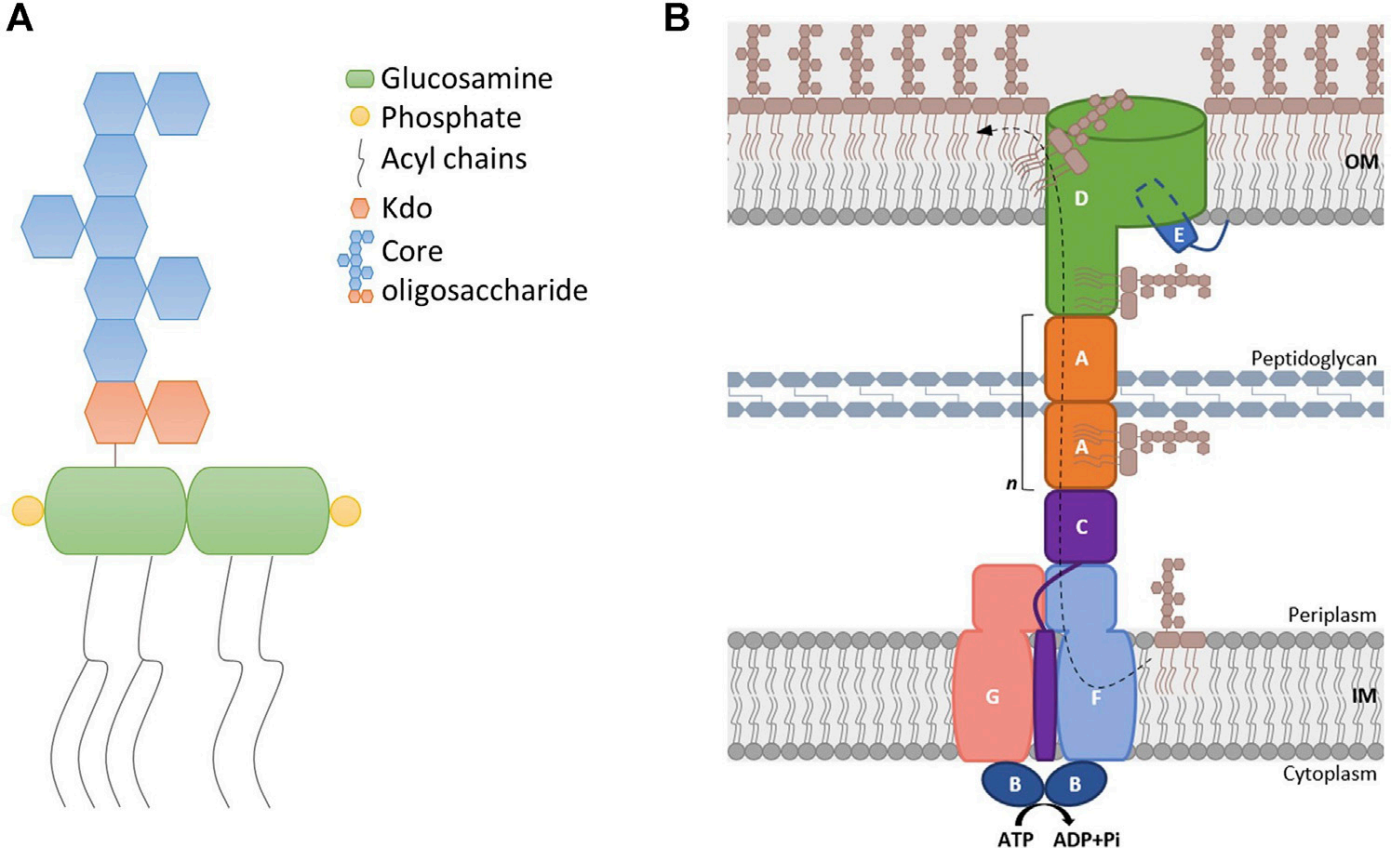
Structure of LPS and the Lpt complex. **(A)** The lipopolysaccharide (LPS) molecule consists of three parts: (i) the lipid A moiety, comprising a phosphorylated glucosamine disaccharide linked to saturated acyl chains that insert into the outer membrane (OM); (ii) the core oligosaccharide formed by non-repeating sugar units; and (iii) the O antigen (not depicted), a long oligosaccharide chain that varies highly between species and strains. **(B)** LPS is extracted from the inner membrane (IM) by the IM complex LptB_2_FG and translocated through the hydrophobic cavity of the protein bridge formed by LptC, LptA, and the N-terminal region of LptD. The C-terminal domain of LptD forms the β-barrel that, together with LptE, inserts LPS into the OM.

LPS is a complex glycolipid with six saturated acyl chains that insert into the OM (Raetz and Whitfield, 2002; Figure 1A). Thanks to its peculiar structure, LPS largely contributes to the permeability barrier function of the OM (Raetz and Whitfield, 2002; Nikaido, 2003).

LPS must be transported across the periplasm, from its final site of synthesis at the IM, to the cell surface. From the IM, the Lpt (lipopolysaccharide transport) transenvelope multiprotein machinery transports LPS to the outer leaflet of the OM. In *Escherichia coli*, the Lpt machine is composed of seven essential proteins (LptA-G) that work together as a single complex to extract LPS from the IM, transport it across the periplasm, and insert it into the OM (Figure 1B) (Sperandeo et al., 2019). The ABC transporter LptB_2_CFG powers the LPS extraction from the IM and its transport across the periplasm to the cell surface (Okuda et al., 2012; Li et al., 2019; Owens et al., 2019). LptA is the soluble protein connecting the IM and OM Lpt sub-complexes (Sperandeo et al., 2007; Suits et al., 2008; Tran et al., 2008; Sperandeo et al., 2011), and exactly how many LptA monomers compose the periplasmic Lpt bridge is not yet known. LPS flows from the periplasmic domain of LptC to LptA, and at the OM, the LptDE translocon receives LPS and inserts it into the outer leaflet (Tran et al., 2010; Freinkman et al., 2011; Dong et al., 2014; Qiao et al., 2014). According to the proposed PEZ model, the IM LptB_2_CFG ABC transporter loads and pushes a continuous stream of LPS monomers into the Lpt bridge in a mechanism resembling a PEZ candy dispenser (Okuda et al., 2016).

The Lpt bridge is formed by the shared β-jellyroll fold architecture of the periplasmic domains of LptF and LptC, the LptA protein, and the periplasmic domain of LptD, which all assemble in a C-to-N terminal configuration (Villa et al., 2013; Tran et al., 2010; Qiao et al., 2014; Suits et al., 2008; Dong et al., 2017; Luo et al., 2017; Okuda et al., 2016). The hydrophobic lumen of the bridge shields the LPS acyl chains from the hydrophilic periplasmic environment. When the LPS transport is compromised via the depletion of Lpt components or via the disruption of protein–protein interactions, LPS molecules accumulate at the outer leaflet of the IM, the Lpt bridge disassembles, and LptA is degraded. The LptA stability has thus been considered a sensor for the correct Lpt complex assembly (Sperandeo et al., 2008; Sperandeo et al., 2011; Moura et al., 2020).

The assembly of the Lpt machinery is carefully monitored by the cells to avoid LPS mistargeting. At the IM, recruitment of LptA by the LptC protein does not occur unless the LptB_2_CFG complex is correctly formed (Villa et al., 2013). The correct formation of the LptDE complex is another key regulatory checkpoint in the assembly of the Lpt machinery. The OM translocon comprises LptD, with an N-terminal β-jellyroll domain and a large C-terminal β-barrel domain, and the lipoprotein LptE buried inside the barrel (Freinkman et al., 2011; Dong et al., 2014; Qiao et al., 2014). For the translocon to be functional, two disulfide bonds connect the N-terminal and the β-barrel domains of LptD, ensuring correct orientation of these domains which is essential for the recruitment of LptA (Ruiz et al., 2010; Freinkman et al., 2012). If the LptDE translocon is not correctly assembled, LptA fails to interact and the β-jellyroll oligomerization process for bridge formation does not occur (Freinkman et al., 2012). This ensures that the Lpt bridges do not couple with defective OM translocons, which would mistarget LPS to periplasm and cause toxicity problems (Chng et al., 2010; Freinkman et al., 2012; Zhang et al., 2013).

We have previously showed that LptA stability in the cell can be considered a sensor of the correct assembly of the Lpt complex (Sperandeo et al., 2011). In this work, we sought to clarify whether the stability of the Lpt complex is also dependent on the presence of its substrate. To this purpose, we analyzed the steady-state level of LptA in cells in which the LPS biosynthesis is blocked. We treated the cells with LPC-058, a compound known to inhibit the activity of LpxC, which catalyzes the first essential step of LPS biosynthesis and we show that not only LptA but also LptD steady-state levels decrease in the cell upon LPS biosynthesis blockage. Since LptD folding and turnover are dependent on two major chaperones/proteases that act at different points of the LptD assembly pathway, namely, DegP and BepA, we investigated their role in the decrease of the LptD level in cells with defective LPS synthesis. Overall, our results provide evidence that LPS is required to keep the machinery assembled, and when absent, due to the interruption of LPS synthesis, the Lpt bridge disassembles, and LptA and its anchor at the OM, the LptD protein, are degraded. Under these conditions, the stability of LptD, but not LptA, is controlled by the periplasmic chaperone/protease DegP.

## Methods

### Bacterial Strains and Growth Conditions

*E. coli* strains used in this work are described in Table 1. Luria-Bertani-Lennox (LB-Lennox) medium (Ghisotti et al., 1992) has been described. 25 mg/ml kanamycin and 0.2% arabinose were added when required. Solid media contained 1% agar. Cells of BB3 strain were grown in 50 ml of LB-Lennox medium supplemented with 0.2% arabinose until OD_600_ 0.2. Then, the cells were harvested, washed three times with fresh LB-Lennox, diluted one hundredfold in fresh medium either with arabinose (permissive condition) or without arabinose (non-permissive condition), and incubated with aeration at 37°C. Cell growth was monitored by OD_600_ measurements.

**TABLE 1:** *Escherichia coli* strains.

Strain	Genotype	Source or reference
BW25113	*lacI* ^*q*^ *rrnB*_T14_ Δ*lacZ*_WJ16_ *hsdR514* Δ*araBAD*_AH33_ Δ*rhaBAD*_LD78_	Datsenko and Wanner (2000)
BB3	BW25113 (*kan araC araB*p*-yrbK*)*1*	Sperandeo et al. (2006)
JW0157	F-, *Δ* (*araD-araB*)*567*, *ΔdegP775:kan*, *ΔlacZ4787* (:rrnB-3), *λ* ^*−*^, *rph-1*, *Δ* (*rhaD-rhaB*)*568*, *hsdR514*	Keio collection, Baba et al. (2006)
JW2479	F-, *Δ* (*araD-araB*)*567*, *ΔlacZ4787* (:rrnB-3), *λ* ^*−*^, *ΔyfgC742:kan*, *rph-1*, *Δ* (*rhaD-rhaB*)*568*, *hsdR514*	Keio collection, Baba et al. (2006)
AMM92	BW25113 Δ*degP*	This work
AMM93	BW25113 Δ*yfgC*	This work

### Strain Construction

*degP* and *bepA* deletion strains were obtained by P1 phage transduction (Silhavy et al., 1984) moving *kan*-marked alleles from the Keio *E. coli* single-gene knockout library (Baba et al., 2006). The *kan* cassette was removed by pCP20-encoded Flp recombinase to generate unmarked deletions (Datsenko and Wanner, 2000). The removal of the *kan* gene was verified by replica plating and colony PCR.

### Growth Conditions of *E. coli* Cells Treated With Several Chemicals/Antibiotics

*E. coli* cells were grown in the LB-Lennox medium. At OD_600_ of 0.1, the cells were treated with 0.031 μg/ml of LPC-058, 20 mM ethylene-diaminetetraacetic acid (EDTA) (Falchi et al., 2018), 0.6 μg/ml polymyxin B, 25 mM ammonium metavanadate (NH_4_VO_3_) (Tam and Missiakas 2005), 30 μg/ml aztreonam (Georgopapadakou et al., 1982), and 20 μg/ml cefsulodin (Curtis et al., 1979). The minimum inhibitory concentration (MIC) for each compound was determined using the broth micro-dilution method (Wiegand et al., 2008; Table 2). Cell growth was monitored by OD_600_ measurements. When treated cells decreased the growth rate compared to untreated cells (indicated in the figures with an arrow), samples for western blot analysis were collected after 5, 10, 20, and 60 min, from both cell cultures, and processed as described in *SDS-PAGE and Immunoblotting*.

**TABLE 2:** Minimum inhibitory concentrations (MICs) of antibiotics/chemicals against *E. coli* strains.

	MIC^a^		
	BW25113	AMM92	AMM93
LPC-058	0.031 μg ml^−1^	0.031 μg ml^−1^	0.031 μg ml^−1^
EDTA	20 mM	NT	NT
Polymyxin B	1.2 μg ml^−1^	NT	NT
NH_4_VO_3_	25 mM	NT	NT
Aztreonam	30 μg ml^−1^	NT	NT
Cefsulodin	20 μg ml^−1^	NT	NT

a MIC, minimum inhibitory concentration; NT, not tested.

### Analysis of Outer Membrane Protein Profiles

The analysis of outer membrane protein (OMP) profiles by SDS-PAGE was performed as described by Yethon et al. (2000) with some modifications. After treatment with LPC-058 as described above, equal numbers of cells (standardized by measuring the optical density at 600 nm) of LPC-058–treated and untreated cultures were harvested by centrifugation. After resuspension in 20 mM Tris buffer (pH 8), the cells were lysed by a passage through a High Pressure Cell Disruptor (Constant Systems) at 21,000 psi and centrifuged (3,500 × g, for 10 min) to remove the cell debris. The total membrane fractions were collected by centrifugation (100,000 × g, for 1 h) and then resuspended in 20 mM Tris (pH 8) 2% Sarkosyl buffer. The Sarkosyl-insoluble OM fraction was collected by centrifugation (100,000 × g, for 1 h), then washed with 20 mM Tris buffer (pH 8), and recentrifuged. The resulting pellet was resuspended in 250 μL of 20 mM Tris buffer (pH 8) and analyzed by sodium dodecyl sulfate–polyacrylamide gel electrophoresis (SDS-PAGE). Input samples from each cell culture were processed to obtain the cell crude extract and analyzed by western blot, as described in *SDS-PAGE and Immunoblotting*.

### SDS-PAGE and Immunoblotting

LptA, LptD, LptB, LptE, LptC, and OmpA steady-state levels were assessed in the BW25113, BB3, and *degP* and *bepA* deletion strains (Table 1) by western blot analysis using polyclonal antibodies against LptA, LptD, LptB, LptE, LptC, and OmpA raised in rabbit. Crude-cell extracts for protein analysis were collected and harvested by centrifugation at the time points indicated in *Growth Conditions of E. coli Cells Treated With Several Chemicals/Antibiotics*. The cell pellets were resuspended in a volume (in mL) of SDS sample buffer equal to 1/24 of the total optical density of the sample. The samples were processed for immunoblotting as previously described (Moura et al., 2020).

### LPS Analysis From Whole-Cell Extracts

Whole-cell extract samples for LPS analysis were obtained as described in the previous section and then separated by 18% Tricine SDS-PAGE (Lesse et al., 1990). Immunodecoration was performed using anti-LPS core WN1 222-5 monoclonal antibodies (Hycult Biotech) at a dilution of 1:1,000 and using anti-LptE antibodies as the loading control. Goat anti-mouse immunoglobulin Alexa Fluor^®^ 788 conjugate and goat anti-rabbit immunoglobulin Alexa Fluor^®^ 688 conjugate (Li-Cor) were used as secondary antibodies at a dilution of 1:15,000.

## Results

### Improper Assembly of the Lpt Machine Causes Selective LptA Degradation

**FIGURE 2:**
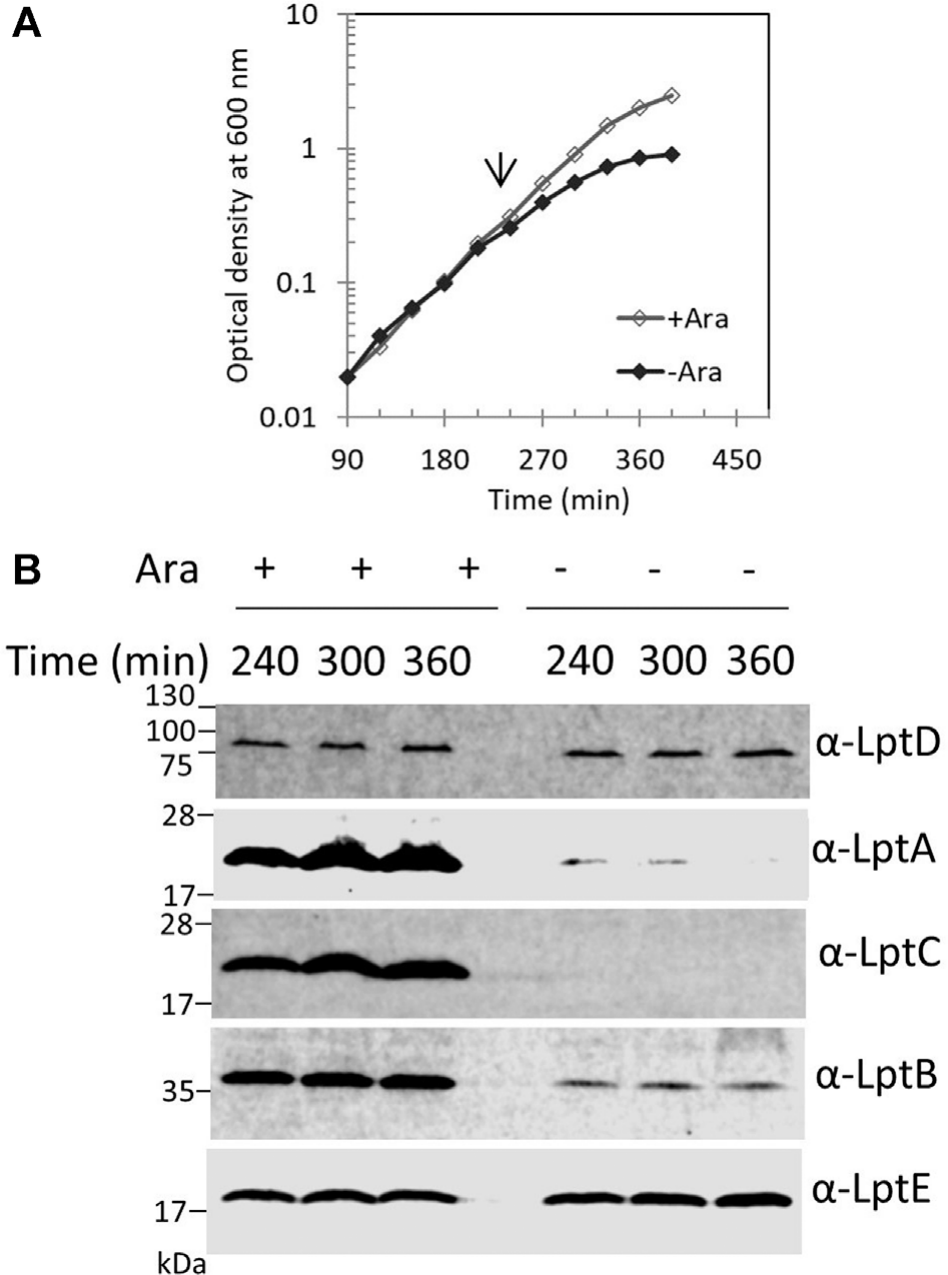
Depletion of LptC does not affect the stability of the IM and OM Lpt sub-complexes. **(A)** Cells of BB3 were grown with 0.2% arabinose until OD_600_ 0.2; the cells were then harvested, washed three times, and resuspended in an arabinose-supplemented (+ Ara) or arabinose-free (no Ara) medium. Samples for analysis of protein stability were collected at the time points indicated. **(B)** Whole-cell extracts were prepared and analyzed by western blot with anti-LptA, anti-LptD, anti-LptC, anti-LptB, and anti-LptE (as the loading control) antibodies. An equal number of cells (0.67 OD_600_) were loaded into each lane. Growth curves and western blot analyses shown are representative of at least three independent experiments.

We previously reported that, in *E. coli* cells, LptA is degraded in the absence of the correct IM and OM docking sites, as depletion of LptC, LptD, or LptE (involved in proper LptD folding) (Chng et al., 2012) results in decreased LptA levels (Sperandeo et al., 2011). To understand whether the lack of LptA interaction with its IM docking site affects the stability of other Lpt proteins, we evaluated the stability of representative components of the IM and OM sub-complexes upon depletion of LptC. For this purpose, the BB3 strain, in which *lptC* expression is under the control of the inducible *araBp* promoter (Sperandeo et al., 2006), was grown under permissive conditions, namely, in the presence of 0.2% arabinose, to the exponential phase and then washed to remove arabinose and shifted to media lacking arabinose to deplete LptC. Samples for protein analyses were taken from cultures grown in the presence or in the absence of arabinose at 240, 300, and 360 min after the shift to non-permissive conditions and then processed for analysis with anti-LptA, anti-LptD, anti-LptC, anti-LptE, and anti-LptB antibodies (Figure 2). We observed a decrease in the steady-state level of LptA but not of other Lpt components. Under non-permissive conditions, LptB displays a lower but stable level than that observed under permissive conditions. It should be noted that, in the presence of arabinose, *lptCAB* are transcribed from the *araB*p promoter. Therefore, under this condition, the level of LptC, LptA, and LptB is higher than that expressed from the native chromosomal promoters (Sperandeo et al., 2011; Martorana et al., 2011). Indeed, under non-permissive conditions, namely, when *lptC* is not transcribed, the expression of the downstream *lptAB* genes is driven by the *lptA* promoter situated within the *lptC* coding region (Sperandeo et al., 2006, 2008; Martorana et al., 2011) resulting in a lower expression of LptA and LptB, and while LptA undergoes degradation, LptB has a lower but stable level (Figure 2). These data suggest that when the Lpt machinery is not functional due to the lack of either IM or OM components, LptA is selectively degraded because of the improperly assembled Lpt complex.

### Blockage of LPS Synthesis Causes LptA and LptD Degradation

**FIGURE 3:**
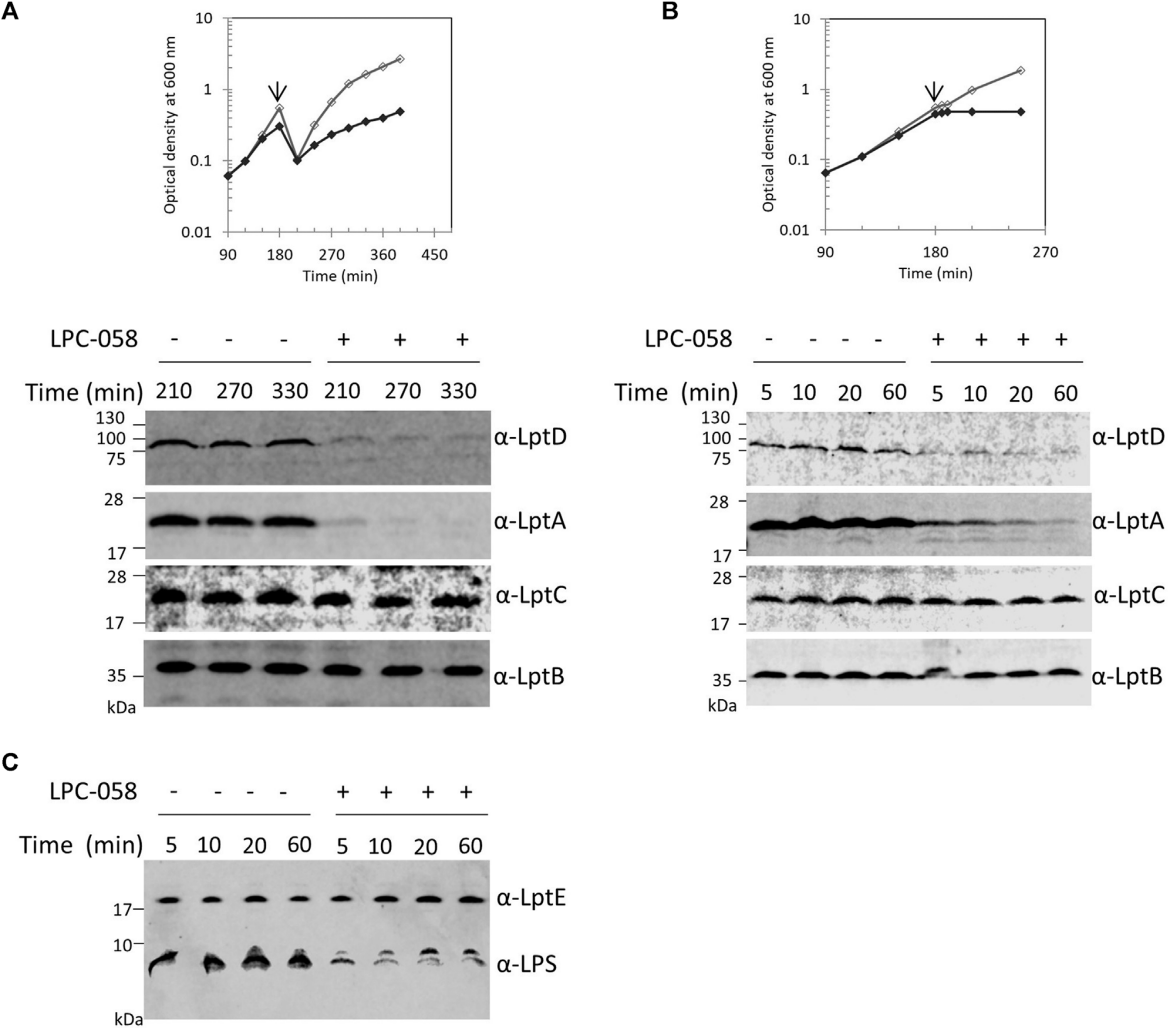
Inhibition of LPS synthesis affects LptA and LptD stability. **(A)** *Escherichia coli* wild-type cells were grown in the LB-Lennox medium. When cells reached OD_600_ of 0.1, they were treated with 1xMIC of LPC-058 (LpxC inhibitor). Untreated cells were used as the control. Cell growth was monitored by OD_600_ measurements. When cultures reached OD_600_ of 1.0, they were diluted 10-fold, and samples for western blot analysis were taken after culture dilution at the indicated times. **(B)** Cells were treated or not treated with LPC-058 as indicated in panel **(A)** but not diluted. When the growth curves of treated and untreated cells separate (arrow), samples for western blot analysis were collected. Time points indicate the minutes after the growth curves separated. **(C)** LPS profiles of wild-type cells treated or not treated with LPC-058. Whole-cell extracts obtained at the same time point as in panel **(B)** were analyzed by western blot with anti-LPS and anti-LptE antibodies. An equal number of cells (0.67 OD_600_ in panels **(A)** and **(B)** and 0.17 OD_600_ in panel **(C)**) were loaded into each lane. Growth curves and western blot analyses shown are representative of at least three independent experiments.

The Lpt machinery functions as a single device, and blocking the LPS transport causes accumulation of LPS at the outer leaflet of the IM (Sperandeo et al., 2008; Sperandeo et al., 2011). To test whether accumulation of LPS at the outer leaflet of IM affects the assembly of the Lpt machinery, and therefore LptA stability, we assessed the LptA steady-state level in cells where LPS synthesis is blocked. For this purpose, we treated wild-type cells with LPC-058. This molecule targets the essential uridine diphosphate-3-O-(R-3-hydroxymyristoyl)-N-acetylglucosamine deacetylase (LpxC), the enzyme that catalyzes the first committed step of the lipid A biosynthetic pathway (Lee et al., 2006; Titecat et al., 2016). We observed that cells treated with 1xMIC of LPC-058 arrested growth and LptA underwent degradation (Figures 3A,B). As expected for inhibitors of LPS synthesis, the total amount of LPS in cells treated with LPC-058 decreases compared to that of non-treated cells (Figure 3C). Surprisingly, the LptD level also decreased. The degradation of LptA and LptD in treated cells was very rapid as at 210 min of growth the two proteins were already undetectable in the crude extracts. To test the kinetics of LptA and LptD degradation, we collected the treated and untreated cells just after the two growth curves separated (Figure 3B). LptA and LptD steady-state levels decreased immediately after the treated cells arrested the growth and are barely detectable after 60 min, suggesting that the two proteins underwent degradation immediately after the LPS level in the cells became insufficient to support growth. Overall, these data suggest that the accumulation of LPS at the IM due to the blockage of transport does not affect the stability and the assembly of the Lpt machinery. Moreover, these results show that, in the absence of the LPS-cargo, both LptA and LptD are degraded, suggesting that, under these conditions, the Lpt machine disassembles.

### Perturbing the LPS Outer Layer Does Not Affect the Stability of the Lpt Complex

Blocking LPS transport results in the alteration of the LPS layer with local loss of OM asymmetry and compensatory accumulation of glycerophospholipids at the outer leaflet of the OM (Nikaido, 2003). We therefore examined whether perturbation of the LPS layer by chemicals or antimicrobial peptides would affect the Lpt complex assembly or stability. We monitored the steady-state level of LptA, LptB, LptC, and LptD in cells treated with ethylene-diaminetetraacetic acid (EDTA), polymyxin B, and ammonium metavanadate (NH_4_VO_3_). The ion chelator EDTA disrupts OM asymmetry by removal of LPS from the OM’s outer layer with consequent flipping of glycerophospholipids from the inner to the outer leaflet of the OM (Nikaido, 1996). Ammonium metavanadate triggers changes in the LPS structure and increases OM permeability (Zhou et al., 1999; Vinés et al., 2005; Tam and Missiakas, 2005; Martorana et al., 2011). Moreover, as metavanadate is an inhibitor of the ATP-binding proteins, it likely interferes with the LptB ATPase (Li et al., 2019). Polymyxin B disrupts the OM by interacting with LPS and displacing cations that are required for the maintenance of membrane integrity (Schindler and Osborn, 1979).

**FIGURE 4:**
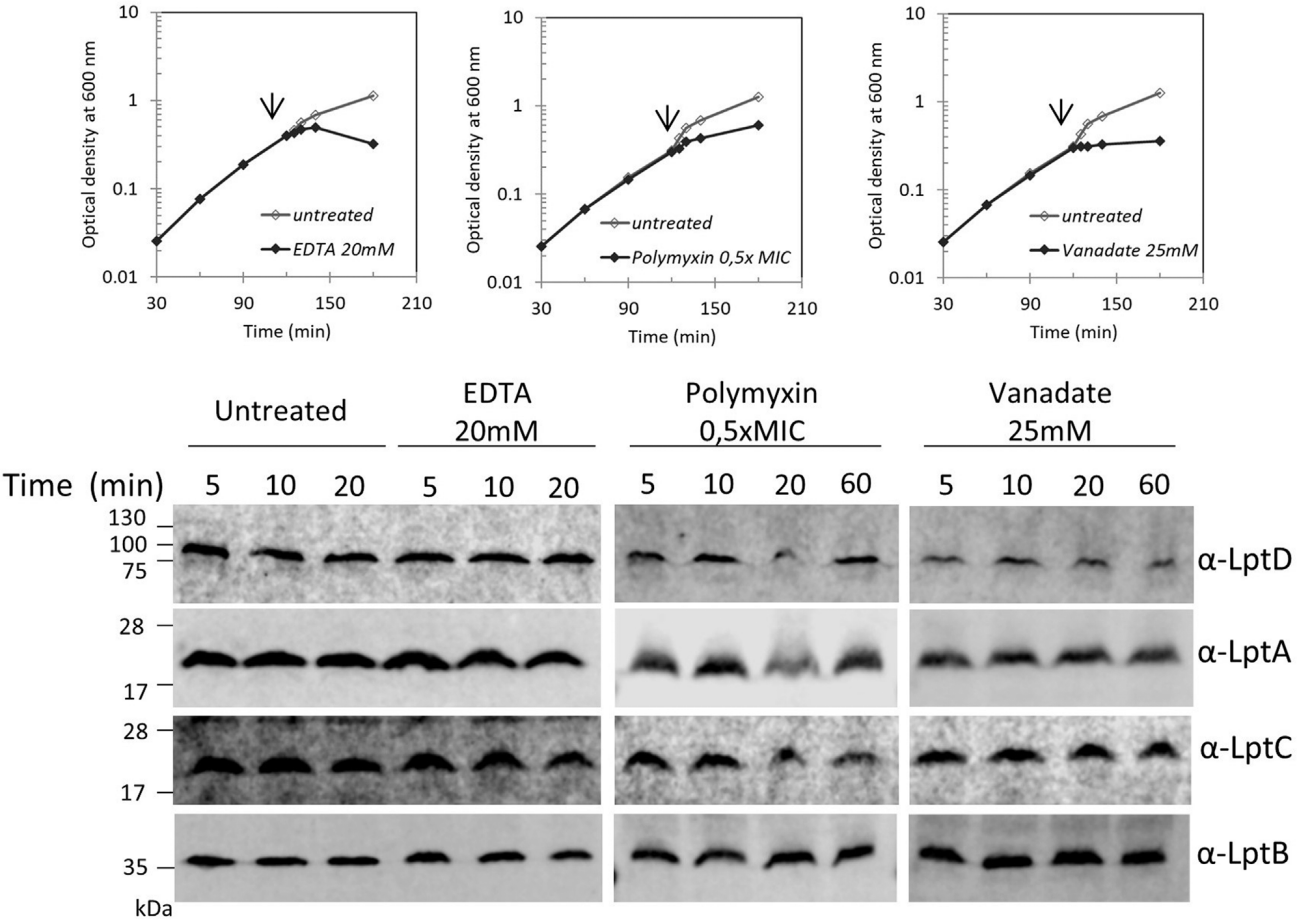
Disrupting the LPS layer at the OM does not affect LptA and LptD levels. *Escherichia coli* wild-type cells were grown in the LB-Lennox medium. When cells reached OD_600_ = 0.1, they were treated with EDTA, polymyxin B, or ammonium metavanadate at the concentration indicated. Untreated cells were used as the control. Cell growth was monitored by OD_600_ measurements. When the growth curves of treated and untreated cells separate (arrow), samples for western blot analysis were collected. Time points indicate the minutes after the growth curves separated. An equal number of cells (0.67 OD_600_) were loaded into each lane. Growth curves and western blot analyses shown are representative of at least three independent experiments.

Surprisingly, none of the compounds affected the steady-state level of LptA, LptB, LptC, and LptD in treated cells in comparison with that of untreated cells (Figure 4). These data suggest that defects in LPS biogenesis, but not perturbations or disruption of the LPS layer, specifically affect the stability and assembly of the Lpt machine.

### Perturbing the Peptidoglycan Does Not Affect the Lpt Complex Stability

**FIGURE 5:**
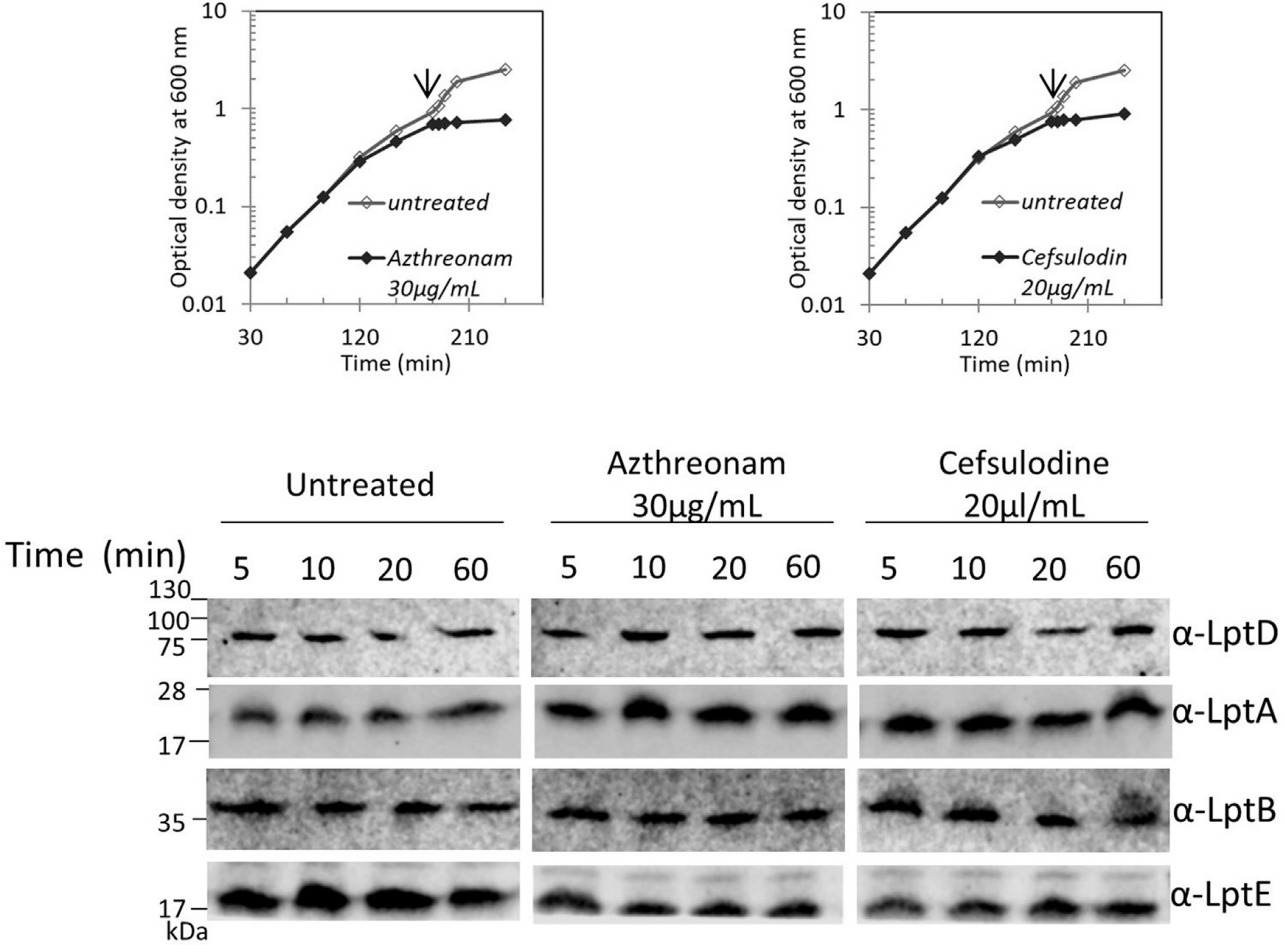
Disturbing the PG layer does not affect LptA and LptD levels. *Escherichia coli* wild-type cells were grown in the LB-Lennox medium. When cells reached OD_600_ = 0.1, they were treated with aztreonam or cefsulodin at the concentrations indicated. Untreated cells were used as the control. Cell growth was monitored by OD_600_ measurements. When the growth curves of treated and untreated cells separate (arrow), samples for western blot analysis were collected. Time points indicate the minutes after the growth curves separated. An equal number of cells (0.67 OD_600_) were loaded into each lane. Growth curves and western blot analyses shown are representative of at least three independent experiments.

We recently showed that defects in LPS biogenesis affect the PG structure, as blocking LPS synthesis or transport activates a PG remodeling program that results in the introduction of 3-3 crosslinks in PG to avoid cell lysis (Morè et al., 2019). These data highlight the importance of synchronizing growth and division of the different cell envelope layers to preserve the structural integrity of the cell (Gray et al., 2015). Based on these data, we tested whether altering the PG layer might compromise the Lpt machinery stability. We therefore treated cells with antibiotics that target PG at different stages of cell growth, namely, aztreonam and cefsulodin. Aztreonam targets PBP3 and affects cell division (Georgopapadakou et al., 1982), while cefsulodin targets PBP1A and PBP1B, the two major bifunctional peptidoglycan synthases, and affects cell elongation (Curtis et al., 1979). Under these conditions, neither LptA nor LptD stability is affected (Figure 5), suggesting that conditions affecting the PG structure and stability do not impair the assembly of the Lpt system.

### The DegP Periplasmic Endoprotease Is Involved in LptD Degradation Upon Blockage of LPS Biosynthesis

**FIGURE 6:**
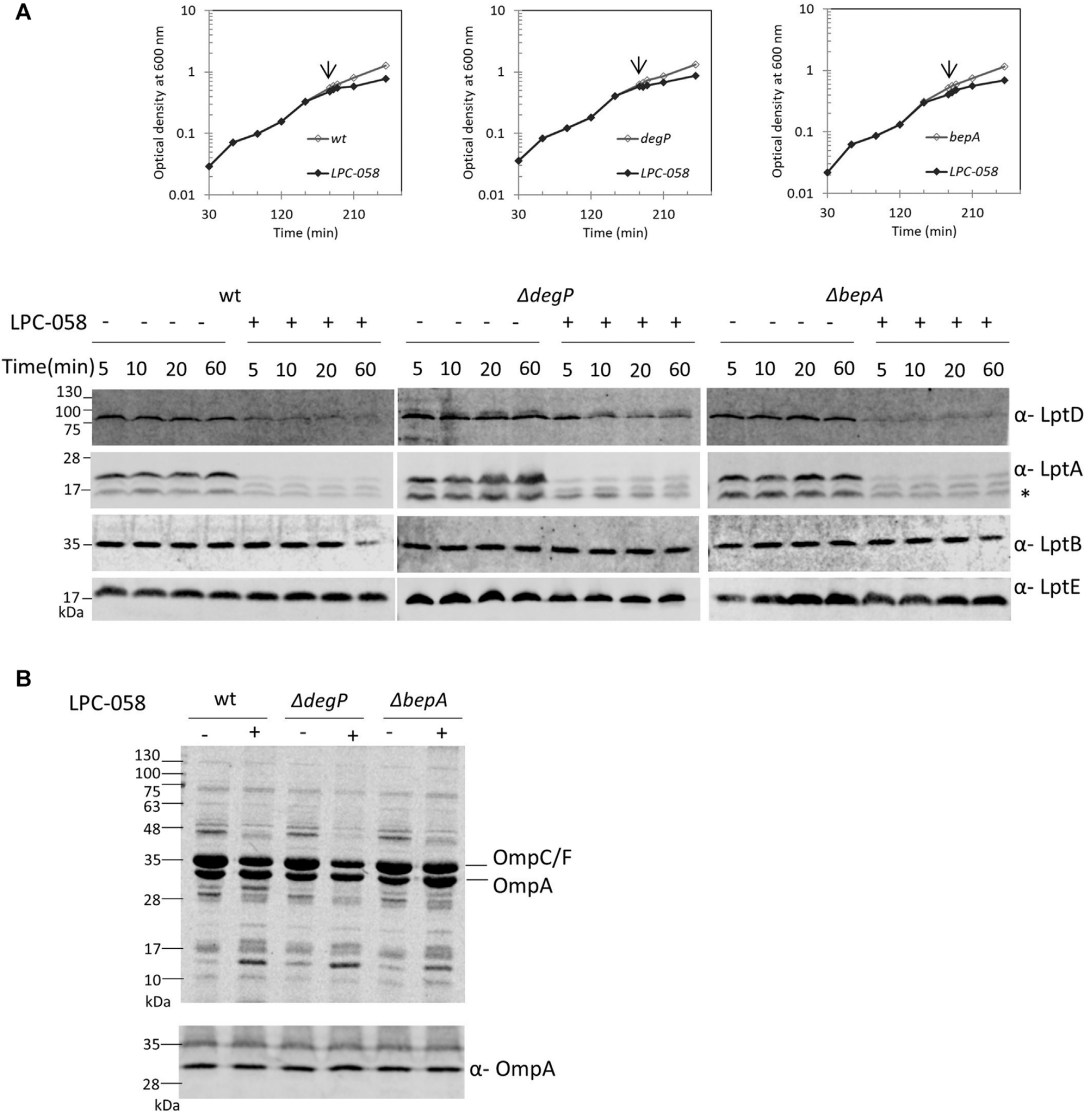
DegP degrades LptD when the LPS biosynthesis is inhibited. *Escherichia coli* wild-type (wt), Δ*degP*, and Δ*bepA* cells were grown in the LB-Lennox medium at 30°C. When cells reached OD_600_ = 0.1, they were treated with 1xMIC of LPC-058 (LpxC inhibitor). Untreated cells were used as the control. **(A)** For the analysis of the steady-state level Lpt proteins, cell growth was monitored by OD_600_ measurements. When the growth curves of treated and untreated cells separate (arrow), samples for western blot analysis were taken. Time points indicate the minutes after the growth curves separated. * indicates the degradation product of LptA. **(B)** OM fractions were purified from cells treated for 2 h with LPC-058. Equal amounts of proteins were loaded (upper panel). Crude extracts of the same cultures were analyzed by western blot, using anti-OmpA antibodies, and equal amounts of cells were loaded (lower panel, 0.67 OD_600_). Growth curves and western blot analyses shown are representative of at least three independent experiments.

LptD stability in the cell relies on two major proteases that act at different points of the LptD assembly pathway: the periplasmic chaperones/proteases DegP and BepA (Soltes et al., 2017; Narita et al., 2013). DegP degrades LptD to prevent initial contact with the β-barrel-assembly machinery (BAM) complex (Tomasek and Kahne, 2021), thus avoiding accumulation of non-functional proteins in the periplasm. BepA, instead, degrades the LptD mutant substrate that has engaged the BAM complex but is not fully functional or not properly folded (Soltes et al., 2017). To determine whether these proteases are involved in the degradation of LptD upon blockage of LPS biosynthesis, we tested LptD stability following LPC-058 treatment in mutants deleted for *degP* and *bepA* (Figure 6A). We found that the deletion of *bepA* does not increase the LptD steady-state level which is comparable to that observed in the non-treated bepA mutant (left panel) and in LPC-058–treated wild-type cells (right panel). On the contrary, the LptD level increases in degP mutant cells treated with LPC-058 (middle panel) compared to that observed in LPC-058–treated wild-type cells. These data suggest that DegP plays a major role in the degradation of LptD upon blockage of LPS biosynthesis. Interestingly, neither single *degP* nor bepA deletions affected LptA degradation.

To address whether LPC-058–induced Lpt protein changes are accompanied by alterations in the protein content of the OM, we examined the OMP profile in wild-type, Δ*degP*, and Δ*bepA* cells grown in the presence or absence of LPC-058. Equal numbers of cells were collected and processed for OM purification with sarkosyl (Yethon et al., 2000) and analyzed by Coomassie-stained SDS-PAGE (Figure 6B, upper panel). The overall OMP profile was very similar among the three untreated strains, and disruption of *degP* gene had little impact on the steady-state levels of OMPs, as already shown by Krojer et al. (2008). The OMP profile shows few minor changes in the LPC-058–treated wild-type cells, where the abundance of the major OMPs is slightly lower in the treated samples and one high-molecular-weight band disappears, whereas one low-molecular-weight band increases. Changes in the protein profile are more evident in the Δ*degP* strain, with the levels of OmpC/F and OmpA decreasing compared to those of wild-type and Δ*bepA*-treated samples.

Finally, we assessed the level of OmpA, a representative major OMP, in whole-cell lysates by western blot. The level of OmpA does not change in wild-type, *degP*, and *bepA* null strains, treated or not with the LpxC inhibitor (Figure 6B, lower panel). This suggests that blockage of LPS synthesis does not affect the overall OMP production and that the observed OMP reduction in the OM of LPC-058–treated cells is likely due to degradation by periplasmic proteases.

## Discussion

Previous work showed that the tight control of LptA level plays a fundamental role in the Lpt machinery assembly and stability (Benedet et al., 2006; Sperandeo et al., 2011; Sperandeo et al., 2019; Moura et al., 2020).

Here, we have further investigated the Lpt assembly requirements. We show that LptA degradation occurs also when LPS synthesis is blocked (Figure 3). Since the LptA level is a proxy for a correctly assembled Lpt complex, we propose that LPS synthesis is fundamental for the assembly of a functional Lpt export machinery. Moreover, using a specific inhibitor of LpxC, the enzyme that catalyzes the first committed step of LPS biosynthesis (Titecat et al., 2016), we discovered that not only LptA but also LptD undergoes degradation, suggesting that a proper LPS biosynthesis is a prerequisite for the assembly of LptD into the OM.

The analysis of the LptA steady-state level upon treatment with agents altering the LPS layer, such as EDTA, polymyxin B, and vanadate, is in line with our hypothesis, as disrupting the LPS layer does not affect the protein level of any of the Lpt components (Figure 4). Interestingly, vanadate treatment, which is known to inhibit LPS transport, as demonstrated by LPS decoration with colanic acid (Martorana et al., 2011), does not affect the LptA steady-state level in the cell. These data suggest that, in the presence of vanadate, the Lpt system is assembled but not functional. This effect could be due to the inhibition of LPS transport caused by vanadate trapped in the LptB_2_FG IM complex (Li et al., 2019). Therefore, upon vanadate treatment, LPS might be jammed in the hydrophobic interior of the Lpt proteins, supporting the idea that the LptA level and, as a consequence, the transenvelope bridge stability correlates with the availability of the LPS substrate.

We recently discovered a PG remodeling pathway essential for survival in cells with defective LPS biogenesis (Morè et al., 2019), thus highlighting the importance of synchronizing growth of these two different cell envelope layers. While it appears that disrupting the LPS biogenetic pathway has a severe impact on the PG structure, the reverse is not true. Indeed, here we show that disturbing PG biosynthesis is not detrimental to the stability of the Lpt system, as the steady-state level of the Lpt proteins is not affected (Figure 5). This result further reinforces the hypothesis that the Lpt system stability depends on the availability of its own cargo. A similar mechanism of substrate-induced assembly of a transenvelope complex is adopted by the HlyA Type 1 secretion system of *E. coli*, where the interaction of the sub-complex composed by the IM ABC transporter HlyB and the membrane fusion protein HlyD with the OM porin TolC is dependent on the presence of the substrate toxin HlyA (Thanabalu et al., 1998). In *E. coli*, additional tripartite (IM, periplasmic, and OM subunits) transenvelope systems exist, which are involved in phospholipid trafficking (Ekiert et al., 2017; Isom et al., 2020). These systems employ two distinct strategies for substrate trafficking: the LetAB system employs the so-called “tunnel” strategy with a single polypeptide bridging the IM and OM, whereas the Mla system employs the “chaperon-like” mechanism in which a soluble periplasmic protein shuttles the substrate from the OM to the IM (Malinverni and Silhavy, 2009; Powers and Trent, 2018). The Lpt system differs in that bridging between the IM and the OM is achieved by interaction of small proteins sharing the β-jellyroll fold architecture (Tefsen et al., 2005; Wu et al., 2006; Sperandeo et al., 2007, 2008; Ruiz et al., 2008; Suits et al., 2008; Okuda et al., 2012) with the LPS substrate playing a key role in maintaining the intermembrane bridge assembled.

The Lpt system assembly is finely regulated at several levels (Sperandeo et al., 2019). Our data suggest the existence of an additional control mechanism occurring when LPS biosynthesis is defective. Indeed, under such conditions, we observed the decrease of both the LptA and LptD levels. It was previously shown that only a correctly folded LptDE OM translocon allows the assembly of a functional Lpt system, since LptD mutant proteins that lack native disulfide bonds do not interact with LptA, thus preventing the formation of the transenvelope bridge (Chng et al., 2012; Freinkman et al., 2012; Okuda et al., 2016). Clearly, the proper maturation of LptD is carefully monitored by the cell and represents a key control step in the formation of the Lpt protein bridge. Here, we show that LPS biosynthesis is also a prerequisite for a proper LptD folding into the OM, and in turn for Lpt system assembly. Alongside the discovery that the OM translocon could communicate with the IM complex to arrest the LPS transport, in OM proteoliposomes that have reached the saturating amount of LPS (Xie et al., 2018), our data unveil that there are multiple control steps of assembling and functioning of the Lpt system.

We show that the stability of LptD in the *degP* null strain upon LPS biosynthesis blockage is increased (Figure 6), suggesting that DegP is the primary protease responsible for degrading LptD when the OM undergoes severe LPS depletion. The biogenesis and assembly of OMPs are tightly regulated processes at transcriptional and post-translational levels (Rhodius et al., 2006; Guisbert et al., 2007; Sklar et al., 2007; Merdanovic et al., 2011). The LptD biogenesis is carefully monitored in the cell, and its folding is assisted by several chaperones (Schwalm et al., 2013; Ruiz et al., 2010). In case of maturation defects, LptD is degraded by DegP to prevent initial contact with the BAM, preventing the unfolded protein from accumulating in the periplasm. In case the defective LptD has engaged the BAM complex, it is degraded by BepA to block the formation of a defective complete β-barrel (Soltes et al., 2017; Daimon et al., 2020). These quality control factors help ensure the integrity of the OM, since defective OMPs could compromise the permeability barrier properties of the OM (Ruiz et al., 2006). We hypothesize that DegP degrades LptD in order to prevent the assembly of LptD in a defective OM. LptD is the OMP with the largest lumen in *E. coli* (Braun and Silhavy, 2002; Botos et al., 2016); hence, its decreased level in a condition in which its lumen is empty (i.e., devoid of its cargo) can be a strategy to counteract the altered permeability of an LPS-depleted OM. Our results underscore the importance of the regulatory network controlling OM homeostasis: while defects in LPS induce the σ^E^ stress response to increase LptD transcription and counteract OMP assembly defects, as LPS is required for efficient OMP folding (Schnaitman and Klena, 1993; Missiakas et al., 1996; Klein et al., 2009), the control of LptD at a post-translational level is a fine-tuning system not only to correct defects in its maturation but also to correct OM permeability defects when the amount of LPS to be transported to the cell surface is limiting.

The LptD level in the cells does not decrease in the absence of DegP, but LptA degradation still occurs even when LptD is correctly maturated and can act as the LptA OM docking site, implying that DegP is not responsible for LptA degradation. We do not know what is the event that triggers LptA degradation; however, our data support that the LptA stability and thus Lpt protein bridge formation are correlated with the availability of LPS to be transported. It has been proposed that the Lpt machinery functions like a PEZ “candy dispenser” where the LPS “candies” are continuously pushed by LptB_2_FG, using the energy provided by ATP hydrolysis, into the periplasmic channel formed by LptCAD up to the LptDE translocon, which finally assembles LPS at the OM (Okuda et al., 2016). The data presented here suggest that the LPS might trigger the assembly of the Lpt system/candy dispenser, and the LPS molecules/candies pushed through the protein bridge might act as a sort of “glue” to maintain the system assembled.

## Data Availability

The original contributions presented in the study are included in the article/Supplementary Material, and further inquiries can be directed to the corresponding authors.
